# Endophytic Fungus from *Opuntia ficus-indica:* A Source of Potential Bioactive Antimicrobial Compounds against Multidrug-Resistant Bacteria

**DOI:** 10.3390/plants11081070

**Published:** 2022-04-14

**Authors:** Wafaa M. Elkady, Marwa M. Raafat, Marwa M. Abdel-Aziz, Arwa A. AL-Huqail, Mohamed L. Ashour, Noha Fathallah

**Affiliations:** 1 Pharmacognosy and Medicinal Plants Department, Faculty of Pharmacy, Future University in Egypt, Cairo 11835, Egypt; welkady@fue.edu.eg (W.M.E.); noha.mostafa@fue.edu.eg (N.F.); 2 Microbiology and Immunology Department, Faculty of Pharmacy, Future University in Egypt, Cairo 11835, Egypt; marwa.mahmoud@fue.edu.eg; 3 Regional Center for Mycology and Biotechnology (RCMB), Al-Azhar University, Cairo 11651, Egypt; marwa2rcmb@yahoo.com; 4 Department of Biology, College of Science, Princess Nourah bint Abdulrahman University, P.O. Box 84428, Riyadh 11671, Saudi Arabia; aaalhuqail@pnu.edu.sa; 5 Department of Pharmacognosy, Faculty of Pharmacy, Ain-Shams University, Abbasia, Cairo 11566, Egypt; 6 Department of Pharmaceutical Sciences, Pharmacy Program, Batterjee Medical College, Jeddah 21442, Saudi Arabia

**Keywords:** *Opuntia ficus-indica*, endophytic *Aspergillus niger*, antimicrobial activity, multidrug-resistant bacteria, benzaldehyde derivatives, cristatumin B

## Abstract

Endophytic *Aspergillus* species represent an inexhaustible source for many medicinally important secondary metabolites. The current study isolated the endophytic *Aspergillus niger* (OL519514) fungus from *Opuntia ficus-indica* fruit peels. The antibacterial activities were reported for both *Aspergillus* species and *Opuntia ficus-indica* fruit peel extract. Extraction of the endophytic fungal metabolites using ethyl acetate and fractionation was performed, yielding dihydroauroglaucin (C1), isotetrahydroauroglaucin (C2), and cristatumin B (C3). Resistant bacterial strains were used to investigate the efficiency of the total fungal ethyl acetate extract (FEA) and the isolated compounds. FEA showed promising wide spectrum activity. (C3) showed excellent activity against selected Gram-negative resistant bacteria; However, (C2) exhibited tremendous activity against the tested Gram-positive resistant strains; conversely, (C1) possessed the lowest antibacterial activity compared to the two other compounds. An in silico virtual molecular docking demonstrated that cristatumin B was the most active antimicrobial compound against the selected protein targets. In conclusion, the active metabolites newly isolated from the endophytic fungus *Aspergillus niger* (OL519514) and present in plants’ waste can be a promising antimicrobial agent against multidrug-resistant bacteria.

## 1. Introduction

*Opuntia ficus-indica* L. (Cactaceae) is known to be one of the most common fruits distributed worldwide. It is also called the prickly pear fruit [1]. Fruits, cladodes, and flowers have long been used in traditional medicine due to their nutritional properties, therapeutic activity, and economic value [2]. Fruit peels may provide valuable new remedies because they fight against insects, bacteria, and other predators, in addition to poor environmental conditions. The antibacterial activity of the prickly pear fruits’ peel was previously reported [3].

Microbial natural products have contributed significantly to the current pharmaceutical industry. Recent advances in bacterial genomics research have proven that the potential of compounds derived from endophytic fungus was much more than previously believed [4]. Ascomycetes and *fungi imperfecti* make up most endophytic fungi. They live inside the host plant in normal circumstances without causing any indications of illness [5]. Endophytes are remarkable in that they can grow in plant tissues, and their presence affects and enhances various critical activities of the host plant; they can stimulate plant growth, activate pathogen defense responses, and act as abiotic stress mediators [6]. Furthermore, because endophytic fungi are associated with living tissues and can help in maintaining the plant’s health, they are not considered saprophytes.

Over the last few decades, bacterial resistance to medicinal medications has become a severe problem. Millions of individuals have died due to the rapid evolution of the resistance mechanisms of bacteria, making it a public health concern. The misuse of antimicrobials is the leading cause of resistance mechanisms. The scientific community has concentrated on finding novel, safer, and more effective pharmaceuticals using natural resources, especially plants and fungi. Natural products or components can act as bacteriostatic and bactericidal agents. These medicines possess considerably fewer undesirable side effects [7,8]. The goal of the current study was to isolate and identify the endophytic fungi existing in the fruit peels of *Opuntia ficus-indica* L., and detect the antimicrobial activity of the fungal secondary metabolites against resistant bacterial strains, followed by isolation and purification of the major constituents accountable for the activity.

## 2. Results

### 2.1. Endophytic Fungus Isolation and Identification

Fungus was isolated from the *Opuntia ficus-indica* peels by repeated culturing on PDA. Based on the morphological and microscopical characteristics, the isolated fungus was preliminary identified as *Aspergillus niger* (*A. niger*). Morphological appearance: within 3–5 days, reveals black, smooth, umbonate colonies with an entire margin and light yellow on the reverse side of the plate. Microscopically it appears as branched, septate hyphae with colorless, smooth conidiophores, biseriate phialides, and spiny black conidia. Further confirmation by PCR amplification and sequencing of an internal transcribed spacer (ITS) rRNA gene techniques was performed. The obtained sequence was submitted to GenBank with an accession number of OL519514. The results showed 99% identity and 98% query coverage with *A. niger*.

### 2.2. Identification of Fungal Ethyl Acetate Extract (FEA) Isolated Compounds

**Figure 1:**
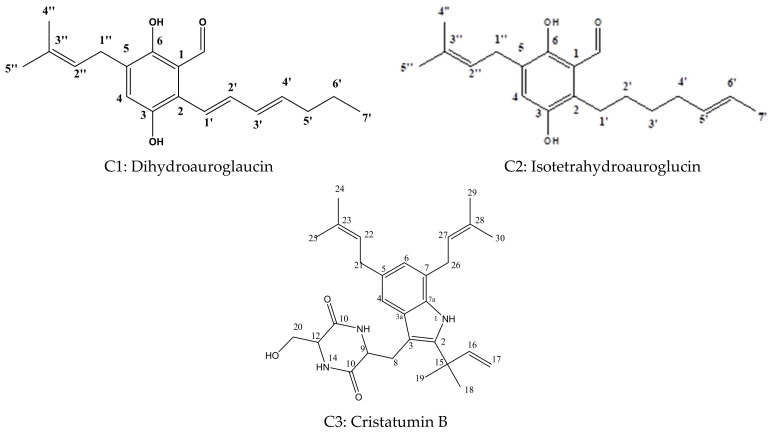
The chemical structures of the isolated compounds from the fungal ethyl acetate extract (FEA).

In order to investigate the active metabolites present in the identified fungus, the purification of crude FEA extracts was performed, and yielded three main compounds (Figure 1), namely, dihydroauroglaucin (C1), isotetrahydroauroglaucin (C2), and cristatumin B (C3). The isolated compounds were identified based on their physical characters and spectral data (^1^H-, ^13^C-NMR), and compared with the literature (Table 1 and Table 2).

#### 2.2.1. Dihydroauroglaucin (C1)

(C1) was obtained as yellowish-brown needle crystals (300 mg). It was isolated and purified on the PuriFlash column from the 50% *n*-hexane-ethyl acetate fraction. Using thin layer chromatography (TLC), it appeared as a single yellow spot in the visible light, turning brown after spraying with 10% sulfuric acid/methanol. The NMR revealed an APT spectrum, carbon signals identified as 5 quaternary, 8 methine, 3 methylene, and 3 methyl carbons, and signals in the olefinic area of the heptyl side chain. Two separate indications at (δ 10.1, s, H-7) and (δ 11.75, s, H-6) and ortho substitution indicated the prenylated benzaldehyde component. The existence of a dimethylallyl isoprene unit at (δ 3.9, d, J = 7.8 Hz, 1H, H-1″) was associated with the olefinic proton (δ 5.9, m, 1H, H-2″), and two singlet deshielded CH_3_ protons (δ 1.6, s, 3H, H-4″) and (δ 1.4, s, 3H, H-5″) were found. Aromatic proton is indicated by developing a singlet signal at (δ 7.03, s, 1H, H-4).

Compound **1**’s NMR data matched and were identical to those previously published [9,10] for dihydroauroglaucin (molecular formula C_19_H_24_O_3_) (Table 1).

#### 2.2.2. Isotetrahydroauroglaucin (C2)

Comparing the spectrum with the previously published data, compound **2** can be identified as isotetrahydroauroglaucin [9,10,11,12,13], with chemical formula C_19_H_26_O_3_ (Table 1). This was isolated as a yellowish powder (100 mg). Like compound **1**, it was purified using the PuriFlash column and eluted from a 95% chloroform/methanol fraction. It has similar physical properties based on TLC; it was observed as a yellow spot in the visible light, turning brownish after spraying with 10% sulfuric acid/methanol. The ^1^H NMR spectrum also revealed similarities to compound **1**, indicating the same basic nucleus with differences at H-1′, 2′, 3′, and 4′ at (δ 2.9, m, 2H, H-1′), (δ 1.6, m, 2H, H-2′), (δ 1.4, m, 2H, H-3′), and (δ 1.3, m, 1H, H-4′), indicating the saturation at the heptyl side chain.

#### 2.2.3. Cristatumin B (C3)

Cristatumin B was obtained as a light-yellow powder (79 mg), isolated from the 20% methanol/chloroform fraction, and purified on a silica column using hexane/ethyl acetate gradient elution. It was observed as one greenish brown spot after spraying with sulfuric acid. The ^1^H and ^13^C NMR spectra (Table 2) comply with the previously published data. It showed a prenylated indole nucleus linked to a methyl diketopiperazine through a methylene bridge, revealing similarities to echinulin, except for the methyl group at C-20, which is replaced with CH_2_OH in cristatumin B (δ 3.9, m, 2H, H-20) [1,14,15,16]. By reviewing the literature, it was observed that this compound was isolated before from many Aspergilli genera. However, to our knowledge, this is the first time it was isolated from the *A. niger* species.

### 2.3. Antimicrobial Activity of the Isolated FEA and Its Compounds

**Figure 2:**
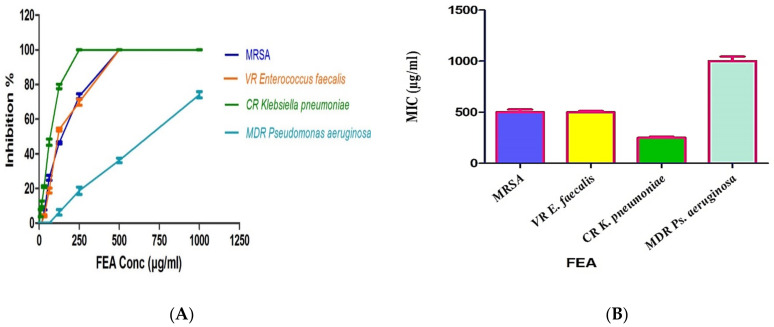
Inhibitory % and MIC values of FEA against different microbial resistance strains. (**A**) % of FEA extract inhibition against MDR bacteria. (**B**) MIC values of the FEA extract. All determinations were carried out in triplicate, and values are expressed as means ± SD.

To achieve the aim of the study, both the total extract FEA and the isolated metabolites were investigated for their antimicrobial activities against different strains of resistant microorganisms. Carbapenem-resistant (CR) *Klebsiella pneumoniae* ATCC BAA-2342, multi drug-resistant (MDR) *Pseudomonas aeruginosa* ATCC -BAA-2111, methicillin-resistant *Staphylococcus aureus* (MRSA) ATCC-700788, and vancomycin-resistant (VR) *Enterococcus faecalis* ATCC BAA-2365. The extract displayed a broad-spectrum antibacterial activity against most of the studied MDR strains with MIC values ranging from 250 to 500 μg/mL. The FEA showed the highest activity against CR *Klebsiella pneumoniae* with MIC = 250 μg/mL, followed by MIC = 500 μg/mL for both VR *Enterococcus faecalis* and MRSA. A diminished activity was noticed against MDR *Pseudomonas aeruginosa* MIC > 1000 μg/mL (Figure 2).

**Figure 3:**
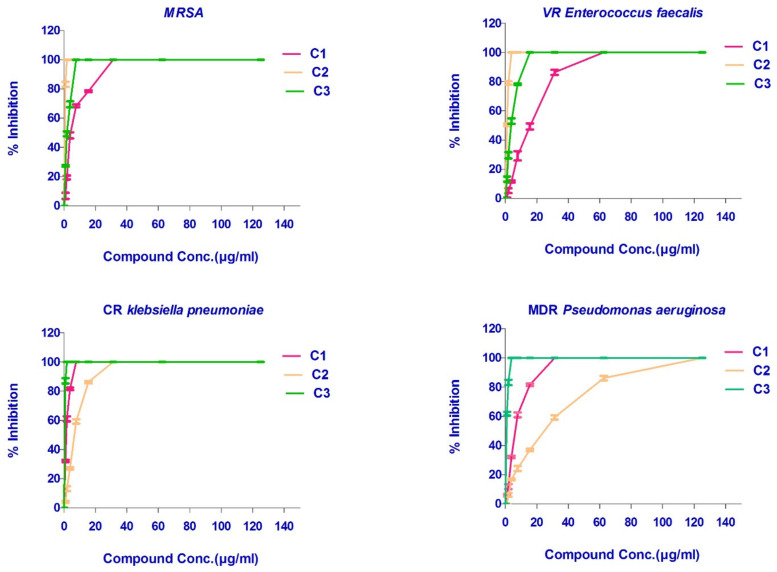
Inhibition % of the isolated compounds against different microbial resistance strains. All determinations were carried out in triplicate, and values are expressed as means ± SD.

The three isolated and identified major phytochemicals were tested for their antibacterial activity. Dihydroauroglaucin (C1) was found to have wide antibacterial action with MICs equal to 7.8, 31.25, and 31.25 μg/mL against CR *Klebsiella pneumoniae*, MRSA, and MDR *Pseudomonas aeruginosa*, respectively. However, it exhibited moderate activity against VR *Enterococcus faecalis* (MIC = 62.5 μg/mL). On the other hand, isotetrahydroauroglaucin (C2) was more prominent against Gram-positive bacteria with MIC values of 1.95 and 3.90 μg/mL against MRSA and VR *Enterococcus faecalis*, respectively. It also exhibited moderate activity against CR *Klebsiella pneumoniae* (MIC = 31.25 μg/mL) and MDR *Pseudomonas aeruginosa* (MIC = 125 μg/mL). Furthermore, cristatumin B (C3) conveyed significant broad-spectrum antibacterial activity. The MIC values were 1.95 and 3.9 μg/mL against the resistant Gram-negative CR *Klebsiella pneumoniae* and MDR *Pseudomonas aeruginosa*, respectively. For Gram-positive VR *Entero. faecalis* and MRSA, MICs were 7.81 and 15.63 μg/mL, respectively (Figure 2 and Figure 3), (Table 3).

### 2.4. Molecular Docking of Isolated Compounds

**Figure 4:**
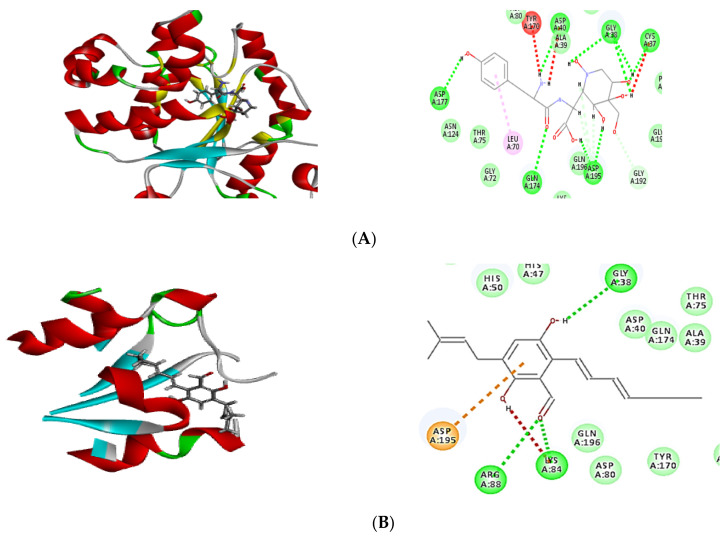

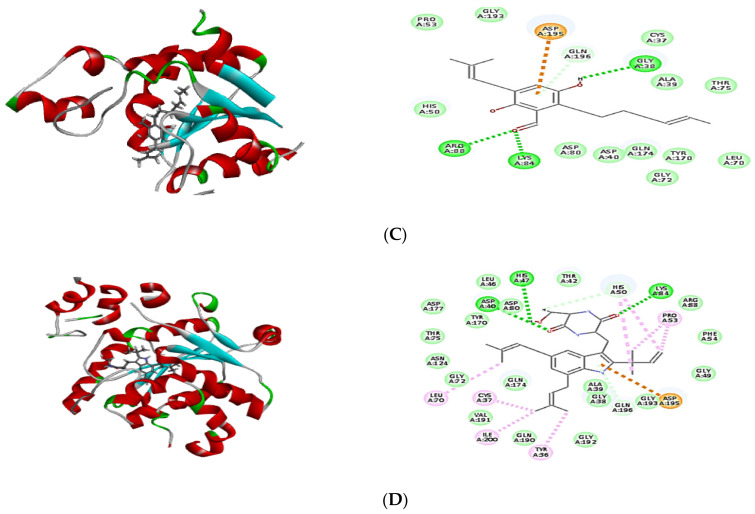
Two-dimensional (2D) and three-dimensional (3D) binding mode of the co-crystalized ligand (629) (**A**), dihydroauroglaucin (**B**); isotetrahydroauroglaucin (**C**); cristatumin B (**D**) with tyrosyl-tRNA synthetase. Residues are labeled with their three-letter amino acid code and their location. Hydrogen-bonding interactions are depicted with a green dashed line between the receptor and the ligand, whereas π–alkyl interactions are represented by a purple dashed line.

**Figure 5:**
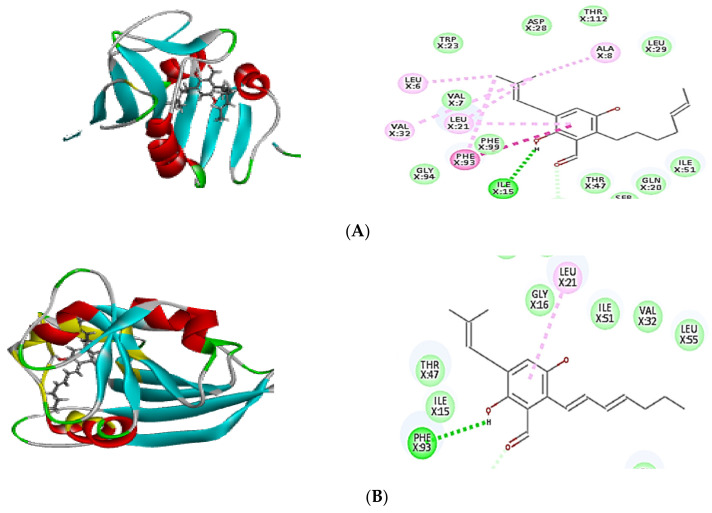

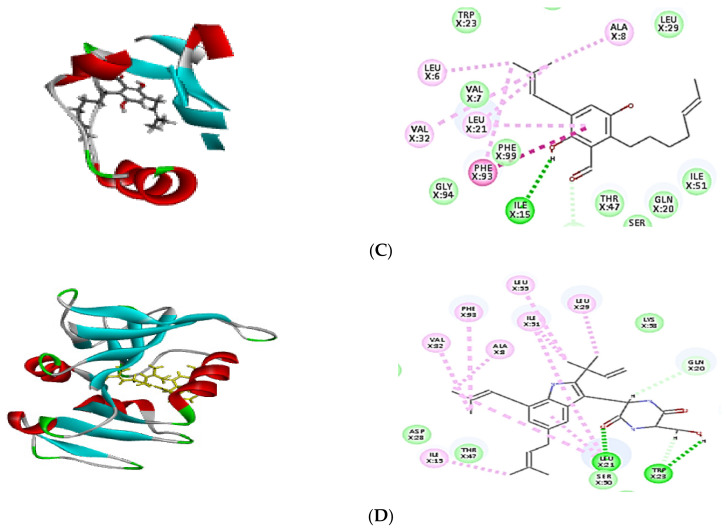
Two-dimensional (2D) and three-dimensional (3D) binding mode of the co-crystalized ligand (Q27) (**A**), dihydroauroglaucin (**B**); isotetrahydroauroglaucin (**C**); cristatumin B (**D**) with dihydrofolate reductase. Residues are labeled with their three-letter amino acid code and their location. Hydrogen-bonding interactions are depicted by a green dashed line between the receptor and the ligand, whereas π–alkyl interactions are represented by a purple dashed line.

**Figure 6:**
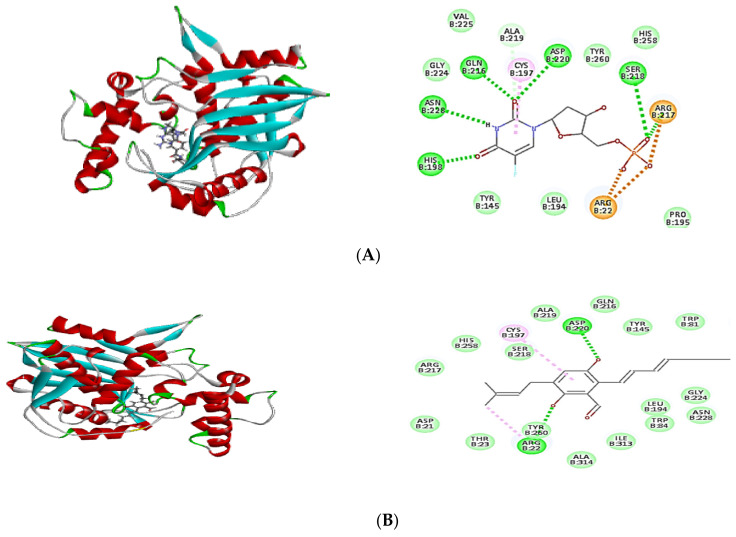

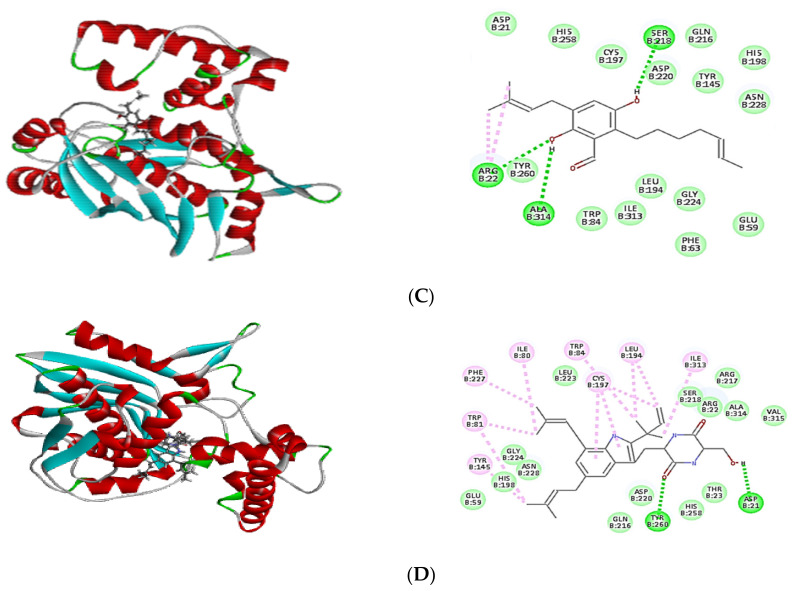
Two-dimensional (2D) and three-dimensional (3D) binding mode of the co-crystalized ligand (FFO) (**A**), dihydroauroglaucin (**B**); isotetrahydroauroglaucin (**C**); cristatumin B (**D**) with thymidylate synthase. Residues are labeled with their three-letter amino acid code and their location. Hydrogen-bonding interactions are depicted by a green dashed line between the receptor and the ligand, whereas π–alkyl interactions are represented by a purple dashed line.

In order to confirm the antibacterial potential, the obtained compounds’ affinity for three different potential antibacterial protein targets was predicted using in silico molecular docking experiments. The results of the molecular docking interactions were presented in Table 4 and Figure 4, Figure 5 and Figure 6. All isolated compounds displayed great affinity to the three tested enzymes; however, cristatumin B (C3) exhibited the maximum affinity.

The potential of the separated phytochemicals under investigation to interact with certain amino acids in the binding location of 1JIJ, 3SRW, and 6qxs receptors via H-bonding and π–π stacking with various amino acids justifies their antimicrobial activity. The co-crystallized ligand was used to compare the docking pattern and docking scores (Table 4). Cristatumin B (C3) exhibited the strongest inhibitory effects, as evidenced by their preferential binding to both the 1JIJ and 3SRW active sites (−63.77 and −55.50, respectively). Each compound’s binding energy and pattern are presented in Table 4 and Figure 4A–C. As a result, the isolated phytochemicals can be considered potential antibacterial agents.

## 3. Discussion

In the twenty-first century, serious infections caused by bacteria resistant to commonly used antibiotics have emerged as a major worldwide healthcare concern [17]. They are not only more severe, but they also demand more time and effort to treat. Changes in antibiotic permeability, changes in target molecules, enzymatic breakdown of medicines, and antimicrobial efflux from the cytosol are all mechanisms of microbial resistance to antibiotics. Bacteria and other microorganisms use these strategies to avoid antibiotic toxicity [18]. The number of new antibiotics created has dropped considerably in recent years. Instead, a reexamination of old drugs is becoming increasingly widespread, and several novel antibiotics have already been discovered [18]. Nowadays, traditional medicinal plants are anticipated to provide more promising antibiotics. Several approaches have been used to find novel compounds with antimicrobial activity or to improve the activity of existing antibacterial drugs from the endophytic fungi inhabiting those plants [19,20]. One of the essential characteristics of endophytes, particularly fungus, is their ability to create a variety of bioactive chemicals that protect plants from different diseases [9].

In this study, *A. niger* was identified as an endophytic fungus from the peels of *Opuntia ficus-indica* fruits. The main goal was to identify the active metabolites from natural microbial sources. *Opuntia ficus-indica* has been widely investigated for its biological antibacterial activity and has been used to treat microbial diseases for hundreds of years [2,3,21,22]. Moreover, the genus *Aspergillus* has a well-established antimicrobial activity and is considered a source of bioactive secondary metabolites with many applications [9,10,11]. Different secondary metabolites extracted from *A. niger* have considerable antibacterial activity against diverse pathogenic Gram-negative and Gram-positive microorganisms such as *Pseudomonas aeruginosa*, *Escherichia coli*, *Proteus mirabilis*, *Klebsiella pneumoniae*, *Staphylococcus aureus*, *Staphylococcus epidermidis*, and Bacillus sp. [19,23,24,25].

Further investigations were undertaken using the *A. niger* secondary metabolites to demonstrate the antibacterial activity using resistant bacterial strains such as MRSA, VR *Enterococcus faecalis*, CR *Klebsiella pneumoniae*, and MDR *Pseudomonas aeruginosa.* MRSA is one of the most common causes of hospital-acquired infections, becoming increasingly difficult to treat due to developing resistance to all antibiotic classes [26]. VR *Enterococci* sp. is an antibiotic-resistant opportunistic microbe obtained from individuals hospitalized for long periods and who have received many regimens of antibiotics [27]. *Klebsiella pneumoniae*, also known as carbapenem-resistant *Klebsiella pneumoniae*, is one of the most frequent carbapenem-resistant bacteria. This is a serious threat because it is linked to higher mortality rates [28]. The infection resulted in a higher mortality rate with a nosocomial *Pseudomonas aeruginosa* related to the organism’s high resistance to antimicrobials, particularly multidrug resistance in healthcare settings [29].

Results demonstrated that the crude FEA of *A. niger* has a broad-antibacterial spectrum activity suppressing various resistant bacteria species with MICs ranging from 250 to 500 μg/mL against different tested strains. Furthermore, fractionation of the FEA results in isolation, purification, and identification of three active compounds. Two prenylated benzaldehyde derivatives were identified as dihydroauroglaucin (C1) and isotetrahydroauroglaucin (C2), in addition to an echinulin derivative, named cristatumin B (C3). All isolated compounds were previously reported from different Aspergillus species [9,15,30], but to our knowledge, this is the first report of isolates from the endophytic fungus *A. niger* that exist in *Opuntia ficus indica* fruits’ peels. Interestingly, all three compounds exhibited broad-spectrum antimicrobial activities, with MICs much less than those obtained by the FEA. The highest activity against drug-resistant Gram-positive bacteria, MRSA and VR *Enterococcus faecalis*, was achieved by isotetrahydroauroglaucin (C2) (MIC < 4 μg/mL), followed by cristatumin B (C3) (MIC < 16 μg/mL) then dihydroauroglaucin (C1) (MIC < 63 μg/mL). By comparison, cristatumin B (C3) revealed the maximum activity against resistant Gram-negative CR *Klebsiella pneumoniae* and MDR *Pseudomonas aeruginosa* with MIC < 4 μg/mL, then dihydroauroglaucin (C1) (MIC < 32 μg/mL), and finally isotetrahydroauroglaucin (C2) (MIC ≤ 125 μg/mL).

Molecular docking is a powerful algorithm for estimating drug option bound configurations and binding affinities [31,32]. For the first time, molecular docking was used to examine the molecular interaction of the three isolated compounds with different drug targets of bacterial metabolic processes. The docking data (Table 4) demonstrated that all compounds had a sufficient molecular basis for interaction with various microibial targets, which may be responsible for the antibacterial activity (Figure 4, Figure 5 and Figure 6). Cristatumin B (C3) was the most potent compound against both tyrosyl-tRNA synthetase (Figure 4) and dihydrofolate reductase (Figure 5); conversely, it was less active against the thymidylate synthase enzyme (Figure 6).

The realized antibacterial activities of the total extract, in addition to the three isolated compounds, may be related to the lipophilic properties. This can be observed through the π–alkyl interactions of the different tested compounds with the target site in the studied enzymes (Figure 4, Figure 5 and Figure 6). Moreover, hydrogen and ionic bonds with different amino acids were also recognized due to the presence of phenolic, alcoholic, and ketonic groups in the isolated phytochemicals. These bonds can effectively interact directly with proteins and consequently interfere with their 3D structure (conformation), resulting in inhibition of the activity [33].

The findings of this study are promising for the discovery of new secondary metabolites with broad-spectrum MDR antimicrobial activity. It might be scaled up to meet new drug development and clinical demands in a cost-effective manner.

## 4. Materials and Methods

### 4.1. Plant Samples

*Opuntia ficus-indica* fruits were purchased from a local market in Egypt in July 2021. Mrs. Therez Labib, former director of the El-Orman Botanical Garden and Consultant of Plant Taxonomy at the Ministry of Agriculture, authenticated the plant sample. A voucher specimen of the plant material (OF 101) was deposited at Future University’s Faculty of Pharmacy’s Department of Pharmacognosy (FUE).

### 4.2. Endophytic Fungus Isolation and Identification

The method described by Hazalin et al. [34] was used to isolate endophytic fungi. In brief, the peels of *Opuntia ficus-indica* were washed several times with sterile water, cleaned for 1 min with 70% ethanol, 1 min with 2% sodium hypochlorite, and washed with sterile water many times before drying in laminar flow. To prevent the bacterial growth on the cut surface touching the agar surface, the cut ends of surface-sterilized segments were removed and aseptically implanted in Potato Dextrose Agar (PDA) (Oxoid, Hampshire, UK) plates enriched with 250 mg/L gentamicin and streptomycin. In addition to the non-inoculated PDA plates that served as a negative control, non-cut surface-sterilized fruits were planted to rule out the presence of epiphytic fungus. The plates were incubated at 25 °C for one to two weeks, and fungal growth was checked daily. The fungus was purified and preserved on PDA slants at 4 °C. This isolation procedure was repeated three times. A conventional taxonomic key was used to identify the isolated fungus morphologically, including colony traits such as texture, form, and color [35]. The mycelia were inspected under a microscope after adding lactophenol cotton blue to a potential fungus cultured for seven days on PDA using the slide culture method [36,37]. Fungus microscopic identification was performed using morphological characteristics of hyphae and conidia.

The universal fungal primer sets of ITS1 (5′-TCC GTA GGT GAA CCT GCG G-3′) and ITS4 (5′-TCC TCC GCT TAT TGA TAT GC-3′) were employed for confirming molecular identification using ITS rDNA sequencing. The PCR product was purified according to the manufacturer’s instructions using a PCR purification kit (Qiagen, Hilden, Germany), followed by sequencing the purified PCR product using an automated sequencer (ABI Prism 377; Applied Biosystems, Foster City, CA, USA). The sequence homologies were analyzed using NCBI-BLAST (http://blast.ncbi.nlm.nih.gov/Blast.cgi). Initial denaturation was at 94 °C for 6 min, followed by 35 cycles of denaturation at 94 °C for 45 s, annealing at 56 °C for 45 s, extension at 72 °C for 1 min, and final extension at 72 °C for 5 min.

### 4.3. Production and Extraction of the Fungus Metabolites

Mass production via solid rice media was used to obtain the secondary fungal metabolites. PDA fungal plugs were inoculated into ten sterile rice jars and cultured at room temperature for 21 days. One-liter Erlenmeyer jars having 100 g rice and 120 mL water were autoclaved at 121 °C for 20 min. After incubation, 600 mL ethyl acetate was added five times for extraction of fungal metabolites until exhaustion, then dried under vacuum, and the residue was kept for biological and chemical investigations [11].

### 4.4. Compounds Fractionation and Purification

A vacuum liquid chromatography (VLC) filled with silica gel 60 mesh was used to separate the FEA extract (12 g) (Merck, Darmstadt, Germany). Elution was carried out utilizing a gradient elution system consisting of *n*-hexane-ethyl acetate from 100 to 0%, followed by chloroform-methanol from 100 to 0%. The resulting fractions were analyzed using a thin layer chromatography plate (TLC), UV with different wavelengths (254 and 365 nm), and vanillin/sulfuric acid reagent. PuriFlash 4100 system (Interchim; Montluçon, France) was used for preparative separations. A mixing HPLC quaternary pump, a PDA-UV-Vis detector 190–840 nm, a fraction collector, and a sample loading module comprised the 25 g flash-NP column (30 μm). The fractions were diluted in dichloromethane DCM (20 mL) before being loaded to the column with 12 g silica. The samples were collected in test tubes; the solvents were evaporated, and the samples were taken to be investigated further. Interchim Software 5.0 was used to control the system and monitor the processes.

### 4.5. Identification of FEA Isolated Compounds

NMR spectra acquired on a Bruker AVANCE HD III 400 MHz spectrometer (Bruker, Fällanden, Switzerland) were used to identify and elucidate the isolated chemicals from the fractions.

### 4.6. Screening of Antimicrobial Activity against Resistant Strains Using Total FEA and Its Purified Compounds

A preliminary screening of the antimicrobial activity of the total FEA extract using reference-sensitive bacterial strains was performed to confirm the previously reported activity of the identified fungus (data not shown). Then, the antimicrobial activity of the FEA extract against resistant bacterial strains was achieved, first followed by the three isolated pure compounds using the same method. The antimicrobial activity was performed by XTT [2,3-bis (2-methoxy-4-nitro-5-sulfo-phenyl)-2H-tetrazolium-5-carboxanilide] reduction assay [9] with minor modifications using four drug-resistant microorganisms: 2 Gram-negative bacteria: CR *Klebsiella pneumoniae* ATCC BAA-2342, and MDR *Pseudomonas aeruginosa* ATCC -BAA-2111; and 2 Gram-positive bacteria: MRSA ATCC-700788, and VR *Enterococcus faecalis* ATCC BAA-2365. Before use, all microbial strains were cultivated overnight at 37 °C in Brain Heart Infusion (BHI) broth (Oxoid, Hampshire UK) and adjusted to 10^6^ CFU/mL.

The total FEA extract and the isolated phytochemicals were serially diluted using DMSO with final concentrations ranging from 1000 to 7.81 μg/mL for the FEA extract and from 125 to 0.98 μg/mL for the isolated phytochemicals. About 50 μL of each dilution was added to the wells of a microtiter plate containing 100 μL sterile Trypticase Soy Broth (TSB), then 50 μL of adjusted microbial inoculum was added to each well. The microtiter plates were incubated in the dark at 37 °C for 24 h. Then, 100 μL of recently prepared sterilized XTT (0.5 g/L in Ringer’s lactate) was added, and the plates were incubated again for 1 h at 37 °C. The calorimetric variance in the XTT experiment was evaluated using a microtiter plate reader (BioTECK, Los Angeles, CA, USA) at 492 nm after three repetitions. Control positive using standard antibiotics and control negative using DMSO were included in each assay. The following formula was used to quantify the reduction in microbial viability caused by inhibition: % of inhibition = [1 − (ODt/ODc)] × 100, where ODt is the mean optical density of wells treated with the tested sample, and ODc is the mean optical density of wells not treated [9,38]. The inhibitory curve was created by plotting the percent inhibition of microbiological growth against sample concentration upon treating the specified compound. The MIC was defined as the concentration of extract or compound that caused a 100% drop in optical density compared to control growth results.

### 4.7. Molecular Docking

The CDOCKER algorithm in Discovery Studio 4.5 was used to assess the likely molecular binding mode between the discovered compounds and various enzymes involved in antimicrobial biological activity (Accelrys Software, Inc., San Diego, CA, USA) [3]. The Protein Data Bank (http://www.rcsb.org/pdb/) was used to obtain the crystal structures of *Staphylococcus aureus* tyrosyl-tRNA synthetase (PDB ID 1JIJ; 3.20 Å), *S. aureus* dihydrofolate reductase (PDB ID 3SRW; 1.7 Å), and *Enteroccocus faecalis* thymidylate synthetase (PDB ID 6qxs; 2.88 Å). Water molecules were removed from the protein, and this was refined. The binding site was identified based on the binding of the co-crystallized inhibitor and the target enzyme. The positive control ligands for all tested enzymes were employed as the positive control ligands. The co-crystallized ligand was removed before docking, and the produced ligands were docked into the protein binding site using rule-based docking. The co-crystallized ligand was removed before docking; subsequently, utilizing rule-based ionization procedures and appropriate settings, the produced ligands were docked into the protein binding site. The interaction energy was computed to study the interaction between the ligand and the receptor. The top 10 ligand-binding poses for each ligand were sorted based on their CDOCKER interaction energies, and the predicted binding interactions were examined before selecting the optimal ligand-binding poses.

## 5. Conclusions

This study represents the first time fruit peels, which are regarded as an industrial waste, were used as a valuable source of endophytic microorganisms in an attempt to valorize their economic value. The isolated *A. niger* is an endophytic fungus from the peel of *Opuntia ficus-indica* fruits, and has important secondary metabolites that possess a broad antibacterial spectrum against the MDR organisms. These compounds may be used alone or in combination with each other, or with currently utilized antibiotics to augment their effects, leading to alleviation of the microorganisms’ resistance. Additional studies are recommended to reveal other types of endophytic fungi hidden inside fruit peels, and their secondary metabolites, mechanism of action, and biological activities.

## Figures and Tables

**Table 1:** ^1^H-NMR and ^13^C-NMR of prenylated benzaldehyde derivatives isolated from *A. niger*.

Compound 1			Compound 2
**Atom No**	**δ_H_, ^1^H-NMR**	**δ_C_ ^13^C-NMR/APT**	**δ_H,_^1^H-NMR**
1	-	130.8 C	-
2	-	127.5 C	-
3	-	145.5 C	-
4	7.0 (s), 1H	125.7 CH	-
5	-	135.8 C	6.36 (m), 1H
6	11.75 (s), OH	158.5 C	-
7	10.1 (s)	196.5 CH	11.83 (s), 1H
1′	6.59 (d, *J* = 15.8 Hz, 1H)	140.7 CH	10.12 (S), 1H
2′	6.46 (dd, *J* = 15.8, 10.1 Hz, 1H)	132.19 CH	2.99 (m), 2H
3′	6.3 (m), 1H	125.49 CH	1.6 (m), 2H
4′	5.47 (m), 1H	119.9 CH	1.4 (m),2H
5′	2.1 (m), 2H	34.9 CH_2_	1.3 (m), 1H
6′	1.3 (m), 2H	29.9 CH_2_	5.3 (m),1H
7′	0.9 (m), 3H	13.40 CH_3_	5.4 (m), 1H
1″	3.34 (d, *J* = 7.8 Hz, 1H)	27.1 CH_2_	1.86 (d, *J* = 1.6 Hz, 1H)
2″	5.3 (m), 1H	121.2 CH	3.44 (d, *J* = 7.6 Hz, 1H)
3″	-	139.8 CH	5.88 (m), 1H
4″	1.7 (s), 3H	17.7 CH_3_	-
5″	1.8 (s), 3H	25.9 CH_3_	1.75 (s), 3H

**Table 2:** ^1^H-NMR echinulin derivative (cristatumin B) isolated from *A. niger*.

Compound 3			
**Atom No**	**δ_H_, ^1^H-NMR**	**Atom No**	**δ_H_, ^1^H-NMR**
1	7.7, br, s, 1NH	16	5.9, m, 1H
2	-	17	5.1, d, *J* = 17.4, 2H
3	-	18	1.2, s, 3H
4	7.3, s, 1H	19	1.5, s, 3H
5	-	20	3.9, m, 2H
6	6.7, s, 1H	21	3.39, m, 2H
7	-	22	5.3, t, 1H
8	3.67, s, 2H	23	-
9	4.4, s, 1H	24	2.1, 2, 3H
10	-	25	2.0, s, 3H
11	6.05, br, s, 1NH	26	3.53, m, 2H
12	4.03, s, 1H	27	5.8, t, 1H
13	-	28	-
14	5.3, br, s, 1NH	29	1.8, s, 3H
15	-	30	1.9, s, 3H

**Table 3:** MIC (μg/mL) of the isolated compounds against resistant bacterial strains.

Compound	MIC (μg/mL)			
	Gram-Positive Strains		Gram-Negative Strains	
	MRSA ATCC-700788	VR *Enterococcus faecalis* ATCC BAA-2365	CR *Klebsiella pneumonia* ATCC BAA-2342	MDR *Pseudomonas aeruginosa* ATCC-BAA-2111
**C1**	31.25	62.5	7.81	31.25
**C2**	1.95	3.9	31.25	125
**C3**	7.81	15.63	1.95	3.9
Vancomycin	1.95	-	-	-
Linezolid	-	1.95	-	-
Colistin	-	-	0.48	3.9

**Table 4:** Free binding energies (∆G) of the identified compounds within the 1JIJ, 3SRW and 6 qxs active site calculated in kcal/mol using Discovery Studio 4.5 adopting rule-based ionization techniques.

Compounds	Binding Energy ∆G (kcal/mol)		
	Tyrosyl-tRNA Synthetase	Dihydrofolate Reductase	Thymidylate Synthase
Co-crystalized ligand (629)	−64.82	-	-
Co-crystalized ligand (Q27)	-	−48.63	-
Co-crystalized ligand (FFO)	-	-	−64.12
Dihydroauroglaucin (C1)	−41.59	−41.77	−42.16
Isotetrahydroauroglucin (C2)	−44.32	−42.75	−43.98
Cristatumin B (C3)	−63.77	−55.50	–54.75

## Data Availability

Data are available in the manuscript.
